# What are people’s perspectives on different labels for neck pain after a motor vehicle crash? A content analysis of randomized study data

**DOI:** 10.1016/j.msksp.2025.103433

**Published:** 2025-10-10

**Authors:** Yanfei Xie, Karime Mescouto, Jenna Liimatainen, Joshua R. Zadro, Tonny Andersen, Michele Curatolo, Genevieve Grant, Gwendolen Jull, Helge Kasch, Joy MacDermid, Eva-Maj Malmström, Sophie Lykkegaard Ravn, Trudy Rebbeck, Anne Söderlund, Julia Treleaven, Hans Westergren, Michele Sterling

**Affiliations:** a RECOVER Injury Research Centre, The University of Queensland, Australia; b NHMRC Centre of Research Excellence: Better Health Outcomes for Compensable Injury, The University of Queensland, Australia; c STARS Education and Research Alliance, Surgical Treatment and Rehabilitation Service (STARS), The University of Queensland and Metro North Health, Brisbane, QLD, Australia; d Centre for Innovation in Pain and Health Research (CIPHeR), The University of Queensland, Brisbane, Queensland, Australia; e Sydney Musculoskeletal Health, Institute for Musculoskeletal Health, Sydney School of Health Sciences, Faculty of Medicine and Health, Sydney Local Health District and The University of Sydney, Sydney, Australia; f Department of Psychology, University of Southern Denmark, Odense, Denmark; g Department of Anesthesiology and Pain Medicine, and CLEAR Center for Musculoskeletal Research, University of Washington, Seattle, United States; h Australian Centre for Justice Innovation, Law Faculty, Monash University, Melbourne, Australia; i School of Health and Rehabilitation Sciences, The University of Queensland, Brisbane, Australia; j Department of Clinical Medicine, Aarhus University, Aarhus, Denmark; k School of Physical Therapy, Western University, London, Canada; l Department of Pain Rehabilitation, Skane University Hospital, Lund, Sweden; m Department of ENT, Clinical Science, Lund University, Lund, Sweden; n Specialized Hospital for Polio and Accident Victims, & Department of Psychology, University of Southern Denmark, Roedovre, Denmark, Odense, Denmark; o Faculty of Medicine Health, School of Health Sciences, The University of Sydney and John Walsh Centre for Rehabilitation Research, Kolling Institute, Sydney, Australia; p School of Health, Care and Social Welfare/ Physiotherapy, Mälardalen University, Vasterås, Sweden; q Rehabilitation Medicine, Department of Health Sciences, Lund University, Lund, Sweden

**Keywords:** Musculoskeletal pain, Diagnosis, Neck injuries, Neck pain

## Abstract

**Background:**

Labels for neck pain after a motor vehicle crash (MVC) influenced recovery expectations and management preferences. Research is needed to understand why these expectations and preferences varied based on the label given.

**Aim:**

To explore how people perceive different labels for neck pain after an MVC.

**Methods:**

We performed a content analysis of qualitative data from a randomised controlled study. 2229 participants with and without neck pain read a vignette describing a patient with neck pain after an MVC, using one of five labels: *whiplash injury*, *whiplash-associated disorder*, *post-traumatic neck pain, neck pain*, or *neck strain*. Participants provided free-text responses on the label’s meaning, associated words/feelings, required health services/treatments, and any confusion about the label.

**Results:**

Compared to *neck strain*, *post-traumatic neck pain*, *whiplash-associated disorder*, and *neck pain* more commonly evoked negative feelings about symptom severity and prognosis (4.7 % for *neck strain* versus 7.2 %–16.0 % for other labels) and psychological distress (7.3 % versus 13.0 %–30.3 %). Regarding treatment preference, *neck pain* most commonly promoted need for passive physical therapies (21.6 %) and imaging (9.8 %), whereas *neck strain* most often promoted need for exercise (11.6 %) and rarely imaging (3.4 %). *Neck pain* was the most confusing label (39.9 %), while *whiplash injury* was the least (14.8 %), with confusion arising from vagueness or a mismatch with diagnostic expectations.

**Conclusion:**

The meanings, feelings and confusions evoked by neck pain labels after an MVC may explain their impact on recovery expectations and management preferences. Clinicians may consider avoid labels associated with negative feelings and lower preferences for guideline-recommended treatments.

## 1. Introduction

Whiplash-associated disorder is the most common clinical presentation following a motor vehicle crash (MVC) injury (Hincapié et al., 2010). Recovery is often prolonged or incomplete, with 32 %–45 % of injured people continuing to experience neck pain one year after an MVC (Al-Khazali et al., 2020). Despite the high prevalence of chronicity, routine imaging and clinical tests do not typically identify any anatomical structural cause for neck pain after an MVC (Curatolo et al., 2011). Ongoing symptoms negatively affect individuals’ quality of life (Tournier et al., 2016), but also contributes to substantial health and economic costs related to treatment, loss of productivity, and third-party insurance claims (Connelly et al., 2006; Bannister et al., 2009).

The vast majority of neck pain following an MVC is of a musculoskeletal nature (Freeman et al., 2020), often referred to as whiplash injury (Teasell et al., 2020). The post-injury symptoms, which may include neck pain, headache, dizziness, arm pain, and sensory disturbances amongst others to various degrees, is termed whiplash-associated disorder (Teasell et al., 2020). The term whiplash was derived from the putative acceleration/deceleration mechanism to the neck that occurs with the MVC (Littlejohn et al., 2014). These terms have met with criticism including perceived negative connotations, lack of pathological accuracy, and frequent association with compensation and litigation (Teasell et al., 2020; Littlejohn et al., 2014; Bostick et al., 2009; Buitenhuis et al., 2008; Bier et al., 2016).

Diagnostic labels of musculoskeletal pain conditions can influence an individual’s expectations of recovery (O’Keeffe et al., 2022a), treatment preferences (O’Keeffe et al., 2022a; Zadro et al., 2021a; Zadro et al., 2022; Haber et al., 2023), perceptions of condition severity (O’Keeffe et al., 2022a; Zadro et al., 2021a; Zadro et al., 2022; Haber et al., 2023), and/or intention to make a compensation claim (Xie et al., 2024). We recently conducted an online randomised study with 2229 participants either with or without self-reported neck pain from the general population to investigate the effect of five labels on recovery expectations and management preferences: *whiplash injury*, *whiplash-associated disorder*, *post-traumatic neck pain*, *neck pain*, and *neck strain* (Xie et al., 2024). We found that participants allocated to *whiplash-associated disorder* or *neck pain* labels had lower recovery expectations than those allocated to *neck strain*. Compared to other labels, the *neck strain* label was also associated with less perceived need for avoiding physical activity, intensive treatments and a second opinion, lower perceived injury seriousness, and/or lower intention to make a compensation claim. While our study showed a quantitative difference in beliefs about neck pain following an MVC based on the label provided, further research is needed to explore the reasons for these differences in people’s beliefs and preferences.

This paper reports the findings from a content analysis of qualitative data collected as part of our online randomised controlled study. The aim was to explore participants’ perceptions of different labels for neck pain after an MVC, focusing on their understanding of the label, the words/feelings and treatment needs the label evoked, and any confusion the label may cause.

## 2. Materials and methods

We conducted a content analysis of qualitative data collected in a 5-arm, online randomised controlled study in a general population with and without self-reported neck pain. Participants provided online consent after reading the online participant information sheet. The study was approved by The University of Queensland Human Research Ethics Committee (2022/HE001479). Because this study was conducted online and did not involve a clinical intervention, it did not fulfil the World Health Organization’s criteria (World Health Organization) for a clinical trial and was therefore not registered prospectively. Nonetheless, we adhered to a structured protocol and reported the findings in accordance with the Checklist for Reporting Results of Internet E-Surveys (CHERRIES) (Eysenbach, 2004).

### 2.1. Participants and recruitment

Participants aged ≥18 years and proficient in reading and writing English from Australia, New Zealand, United Kingdom, Canada, and United States were recruited through Qualtrics (www.qualtrics.com) from September 16, 2022 to October 04, 2022. Qualtrics is a market research company that recruits nationally representative panels of individuals who have consented to participate in research studies. Details regarding Qualtrics’s sampling and recruitment strategies are reported elsewhere (Qualtrics, 2019). Two groups of participants were recruited: (1) adults with no history of neck pain; (2) adults with a history of neck pain, either pain-free or experiencing neck pain at the time of participation.

### 2.2. Data collection

Participants provided information on sociodemographic details, health literacy, prior knowledge of neck pain following an MVC, feeling of anxiety and depression in the previous week, and where applicable, neck symptoms and healthcare use. Details regarding how these data were collected are described elsewhere (Xie et al., 2024).

Participants then read a vignette describing a patient with neck pain of a musculoskeletal nature after an MVC who consulted a healthcare provider (Box 1). The vignette was developed based on previous studies (Van Eck, 2008; Clough et al., 2015; Ferrari et al., 2010) and were further refined with input from clinicians experienced in managing and/or researching neck pain after an MVC. After reading the vignette, participants were randomly assigned to one of the five diagnostic labels in a 1:1:1:1:1 ratio using the web-based, randomiser function in Qualtrics. Each label was accompanied by a brief explanation:

- *Whiplash injury*. It is an injury to the soft tissues in the neck, often caused by a sudden backward and/or forward motion of the head and neck.
- *Whiplash-associated disorder*. Whiplash-associated disorder refers to symptoms such as neck pain, headache and dizziness that may be present after a sudden backward and/or forward motion injury to the head and neck.
- *Post-traumatic neck pain*: It is an injury to the soft tissues of the neck triggered by a traumatic injury.
- *Neck pain*. You have pain, tension, soreness, stiffness in the neck, most likely involving the soft tissues of the neck.
- *Neck strain*. Many soft tissues such as muscles, ligaments and tendons support the neck. You have strained some of these soft tissues.

After participants received their label, they read the same message from a healthcare provider, which aimed to reassure them that their condition was not serious and encouraged them to return to daily activities (Box 1). Participants then provided free-text responses to the following questions in text boxes without word limits:

1. What does the term “[one of the five labels]” mean to you?
2. When you hear the term “[one of the five labels]”, what words or feelings does this make you think of?
3. What treatment(s) (if any) do you think a person with “[one of the five labels]” needs?
4. Is there anything confusing about the diagnosis (“[one of the five labels]”) given by the healthcare provider in the scenario?

Finally, participants were asked to complete four manipulation check questions to ensure data quality and validity. These included two compliance questions to assess participants’ understanding of the survey instructions and two vignette-related questions to evaluate comprehension of the vignette. Details of these questions are reported elsewhere (Xie et al., 2024). Participants who answered any of these questions incorrectly were considered to have failed the manipulation check and were excluded from the data analysis.

### 2.3. Data analysis

We conducted a content analysis on free-text responses to the four questions, combining both quantitative and qualitative methods to systematically examine the content and frequency of categories to be reported (Drisko et al., 2016). This method, used in previous similar studies (Zadro et al., 2021b; O’Keeffe et al., 2022b), involves coding key words or phrases, summarising similar codes into categories, and connecting related categories into broader themes (Drisko et al., 2016). The analysis was underpinned by a post-positivist paradigm, where reality is considered objective and measurable, but with a recognition that experiences and contexts play a role on peoples’ values and experiences (Lincoln et al., 2013). In the context of this study, a post-positivist paradigm means that the truth about labels is considered objectively while acknowledging participants’ unique perspectives of these labels. We used an inductive approach to this content analysis, where codes were not predetermined but developed during data analysis. Therefore, we minimised subjective interpretation and drew meaning directly from participants’ responses.

A coding framework was developed iteratively for each question by three authors - YX with a physiotherapist background and experience in content analysis, JL with an exercise physiologist background and experience in qualitative research, and KM with a physiotherapist background and expertise in qualitative research. They familiarised themselves with the data, randomly selected 25 % of all responses from each labelling group for each of the questions and developed an initial coding framework for each question. Discrepancies were resolved through discussions. Then, one author (JL) applied the consensus coding framework to the remaining data, with another author (YX) checking for accuracy and consistency. Following this, three authors (YX, JL, and KM) collaboratively refined all codes and organised similar codes into categories and themes, incorporating feedback from an additional author (MS, a physiotherapist and senior researcher with extensive expertise in neck pain after an MVC). During this process, we established a set of coding rules: 1) uninformative or unclear responses (e.g. “Na”, “Nil”, “Do not remember”) were not coded; 2) non-English language responses were excluded because one of the inclusion criteria was proficient in writing English; 3) responses could be assigned to multiple categories/themes, as appropriate, to fully represent the content of the responses; 4) all categories/themes were included in the results, irrespective of how frequently they were mentioned.

Following the method of previous studies (Zadro et al., 2021b; O’Keeffe et al., 2022b), we used descriptive statistics (counts and percentages) to summarise the content analysis results.

## 3. Results

Of the 2262 eligible participants randomised, 2227 (98.5 %) were included in this content analysis after excluding 33 with missing data on outcome measures and 2 with non-English responses.

Participants (46.7 ± 17.5 years; 72.4 % females) across groups had similar sociodemographic characteristics, health literacy, and feelings of anxiety and depression in the previous week, with details reported elsewhere (Xie et al., 2024). Among those with previous or current neck pain (n = 1466; 65.8 %), similar proportions in each group experienced neck pain at the time of participation (39.4 %), had an MVC history (15.7 %), received a specific diagnosis (16.2 %), had imaging (34.0 %) and had taken sick leave (31.2 %). Among participants with current neck pain, the mean (±SD) pain intensity in the previous week was 5.1 (±1.7) on a scale from 0 to 10. Approximately half of these participants experienced neck pain for more than 12 months (51.7 %), which moderately to extremely interfered with daily activities (50 %).

We analysed 8782 responses across four questions (2214 from Question 1, 2209 from Question 2, 2201 from Question 3, and 2158 from Question 4), excluding uninformative responses (15 from Question 1, 18 from Question 2, 28 from Question 3, 69 from Question 4). Themes, categories, detailed descriptions, and examples of participants’ responses for each question are presented in Tables 1–4. Main findings from each question are summarised below.

### 3.1. Question 1: What does the term ‘[one of the five labels]’ mean to you?

For this question, 16 categories and 6 themes were developed: ‘injury mechanisms’, ‘physical symptoms and function’, ‘divergent expectations’, ‘psychosocial implications’, ‘management strategies’, and ‘diagnostic clarity and significance’ (Table 1).

#### 3.1.1. Injury mechanisms

**Fig. 1.**
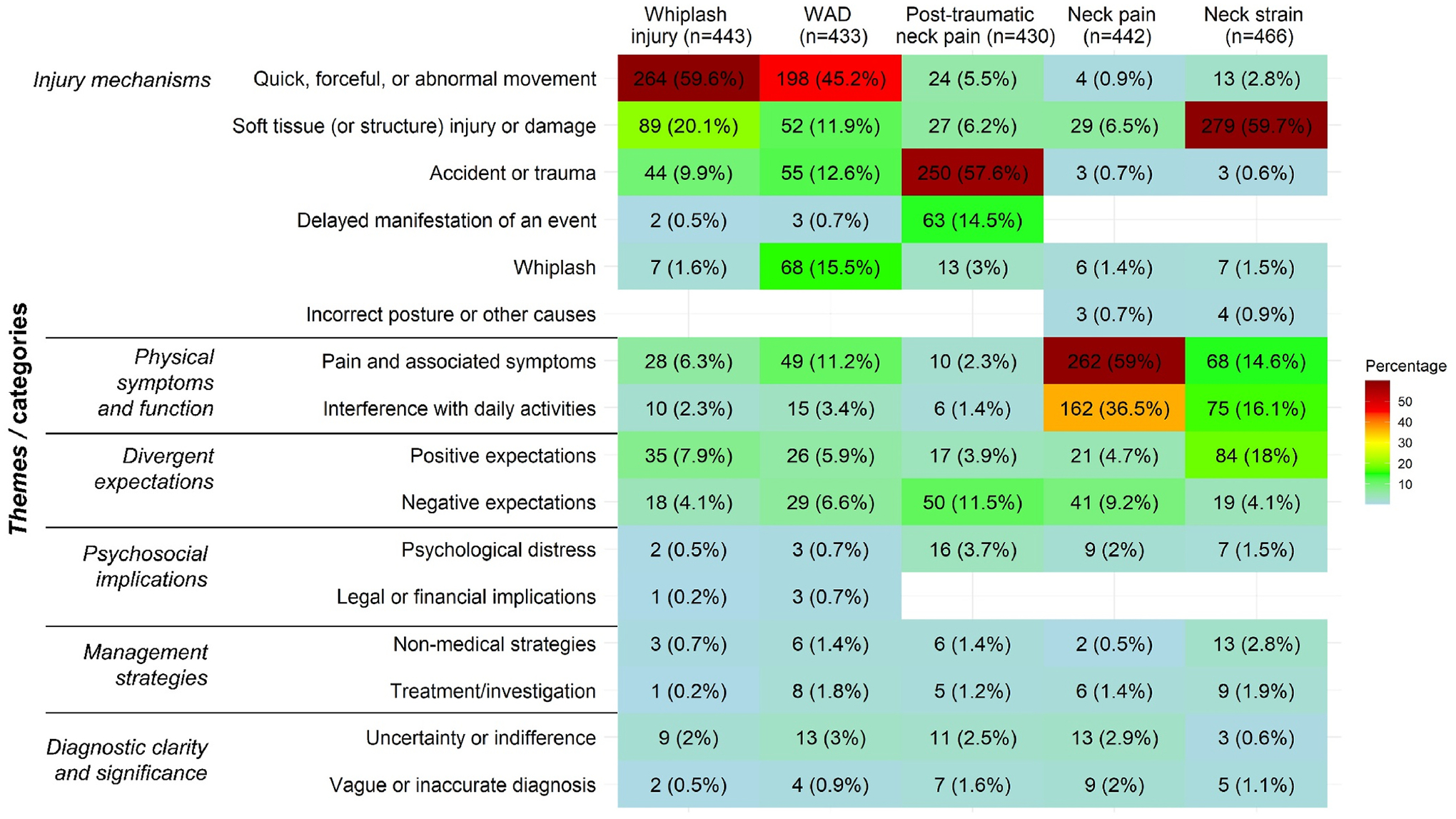
Heatmap shows the frequency of the responses consistent with the themes and categories across label groups for the question: “What does the term ‘[one of the five labels]’ mean to you?”. WAD = Whiplash-associated disorder.

Most participants related the meaning of their assigned label to injury mechanisms (contributing factors of the pain). For example, 59.6 % and 45.2 % of those allocated to *whiplash injury* and *whiplash-associated disorder*, respectively, interpreted it as pain or an injury from quick, forceful, or abnormal movements. 57.6 % of those allocated to *post-traumatic neck pain* interpreted it as pain or an injury sustained from ‘accident or trauma’, while 14.5 % referred to a delayed manifestation after an event (e.g. MVC). *Neck strain* was most frequently interpreted as soft tissue (or structure) injury or damage (59.7 %). Interpretations for these labels were consistent with the brief explanation provided in the vignette. Among participants allocated to the three non-whiplash labels (*post-traumatic neck pain*, *neck pain* and *neck strain*), 1.4 %–3 % still associated them with ‘whiplash’. Few participants (<1 %) allocated to *neck pain* and *neck strain* linked these labels with ‘incorrect posture or other causes’ (Fig. 1).

#### 3.1.2. Physical symptoms and function

Another way that participants referred to the meaning of the labels was by describing the physical symptoms and interference with daily activities. Participants allocated to *neck pain* most frequently associated it with pain and related symptoms (59.0 %). Participants allocated to *neck pain* (36.5 %) and n*eck strain* (16.1 %) more often mentioned interference with daily activities compared to those allocated to other labels (1.4 %–3.4 %).

#### 3.1.3. Divergent expectations

For some participants, their assigned label meant positive or negative expectations about symptom severity, prognosis, or treatment efficacy. Positive expectations were most often expressed by participants allocated to *neck strain* (18.0 %) and least often expressed by those allocated to *post-traumatic neck pain* (3.9 %) and *neck pain* (4.7 %). In contrast, participants assigned to *post-traumatic neck pain* (11.5 %), *neck pain* (9.2 %), and *whiplash-associated disorder* (6.6 %) more often expressed negative expectations than those assigned to *whiplash injury* (4.1 %) and *neck strain* (4.1 %).

#### 3.1.4. Psychosocial implications

A small proportion (0.2 %–3.7 %) of participants referred to psychological distress and legal or financial implications when describing their meaning of the label (Fig. 1). Legal or financial implications were only mentioned by few participants allocated to *whiplash injury* (0.2 %) and *whiplash-associated disorder* (0.7 %).

#### 3.1.5. Management strategies

Few participants referred to management strategies required or actions they would take in response to receiving the label, interpreting the label as a condition that required non-medical care strategies such as time, rest, returning to usual activities, or being cautious for recovery (0.5 %–2.8 %), and/or necessitated treatment or investigation (0.2 %–1.9 %).

#### 3.1.6. Diagnostic clarity and significance

Different from other themes, here participants expressed concerns and uncertainty about their assigned label. 0.5 %–2 % mentioned their label vague or inaccurate, while 0.6 %–3 % felt uncertainty or indifferent towards it (Fig. 1).

### 3.2. Question 2:When you hear the term ‘[one of the five labels]’, what words or feelings does this make you think of?

**Fig. 2.**
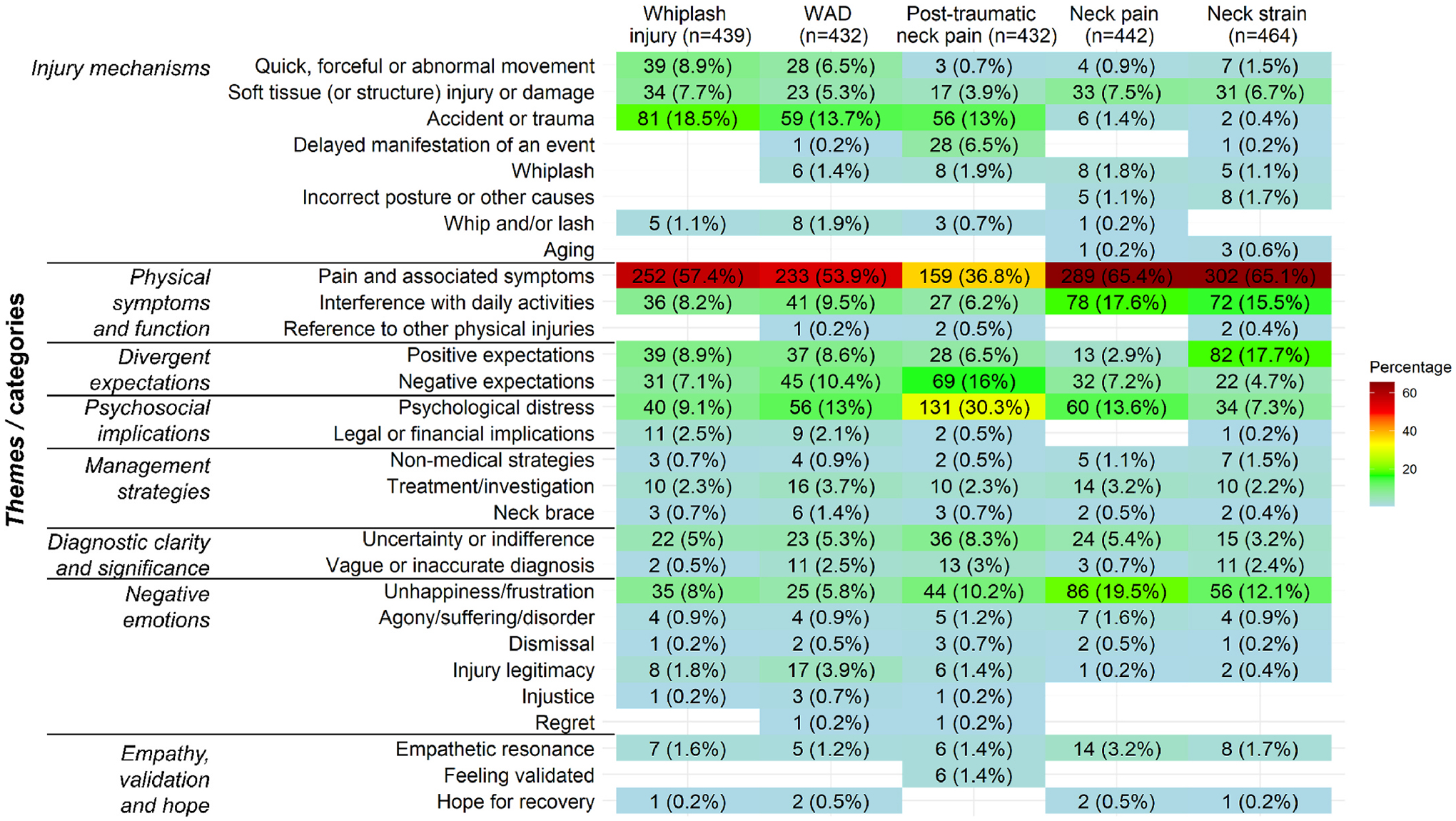
Heatmap shows the frequency of the responses consistent with the themes and categories across label groups for the question: “When you hear the term ‘[one of the five labels]’, what words or feelings does this make you think of?”. WAD = Whiplash-associated disorder.

For this question, 29 categories and 8 themes were identified. Of these, 16 categories and 6 themes overlapped with findings from Question 1 (Fig. 2). Two new themes related to feelings were identified: ‘negative emotions’ and ‘empathy, validation, and hope’ (See their explanations in Table 2).

In contrast to Question 1, across all label groups, responses from Question 2 showed that fewer participants (≤18.5 %) mentioned words consistent with the theme of ‘injury mechanisms’, while more participants (36.8 %–65.4 %) mentioned ‘pain and associated symptoms’ under the theme ‘physical symptoms and function’. Two new categories were identified within the theme ‘injury mechanisms’: ‘whip and/or lash’ and ‘aging’ (Table 2). 0.2 %–1.9 % of participants assigned to *whiplash injury*, *whiplash-associated disorder*, *post-traumatic neck pain*, and *neck pain* associated their label with ‘whip and/or lash’, referring to visual and auditory aspects such as the swift motion of a whip and the sound of a whip cracking. A few participants linked *neck pain* (0.2 %) and *neck strain* (0.6 %) to an aging process. Within the theme ‘physical symptoms and function’, a new category – ‘reference to other physical injuries’ – was developed, with a few participants given the labels *whiplash-associated disorder* (0.2 %), *post-traumatic neck pain* (0.5 %) and *neck strain* (0.4 %) linking their label to other physical injuries such as concussion, football injuries, or ankle sprain.

Similar to Question 1, responses from Question 2 showed that, among the five labels, *neck strain* (17.7 %) most frequently evoked positive expectations, while *post-traumatic neck pain* (16.0 %) and *whiplash-associated disorder* (10.4 %) elicited negative expectations (Fig. 2). The labels *post-traumatic neck pain* (30.3 %), *whiplash-associated disorder* (13 %) and *neck pain* (13.6 %) more frequently evoked feelings of psychological distress than *neck strain* (7.3 %). Additionally, compared to other labels, *whiplash-associated disorder* (1.4 %) mostly frequently made participants think about ‘neck brace’ – a new category identified within the theme ‘management strategies’.

The new theme ‘negative emotions’ suggested that labels evoked diverse negative feelings, including unhappiness/frustration, suffering, injury legitimacy, dismissal, injustice, and regret (Table 2). Unhappiness/frustration was most commonly linked to *neck pain* (19.5 %), and least to *whiplash-associated disorder* (5.8 %). Despite infrequent overall (0.2 %–3.9 %), feelings of injury legitimacy, injustice and regret were more often or exclusively associated with *whiplash injury*, *whiplash-associated disorder* or *post-traumatic neck pain* (Fig. 2). Few participants across groups (0.2 %–0.7 %) felt dismissal, possibly due to the label itself or how it was delivered. Conversely, a small proportion (0.2 %–3.2 %) associated their assigned label with empathy for people with similar injuries, feeling validated and believed, and hope for recovery.

### 3.3. Question 3:What health service(s) /treatment(s) (if any) do you think a person with ‘[one of the five labels]’ needs?

For this question, 20 categories and 6 themes were developed: ‘healthcare professionals and services’, ‘passive treatments’, ‘exercise and advice’, ‘no treatment, non-medical, or alternative care’, ‘investigation and monitoring’, and ‘uncertain about treatment direction and efficacy’ (Table 3).

#### 3.3.1. Healthcare professionals and services

This theme captures a range of healthcare professionals and services mentioned by participants, from allied health professionals to general and specialist medical services. Physiotherapy was most frequently mentioned by all participants, especially those allocated to *post-traumatic neck pain* (51.3 %) and *whiplash-associated disorder* (49.9 %) compared to other labels (40.1 %–42.4 %). Participants assigned to *neck pain* (16.2 %) and *whiplash-associated disorder* (12.8 %) more frequently expressed the need for chiropractic care than those allocated to other labels (8.8 %–9.5 %). Few participants across all label groups expressed need for psychological support (0.2 %–2.1 %), care from other allied health professionals or a nurse (1.4 %–3.2 %), a general practitioner (4.3 %–7.5 %) or a specialist (0.9 %–1.9 %).

#### 3.3.2. Passive treatments

**Fig. 3.**
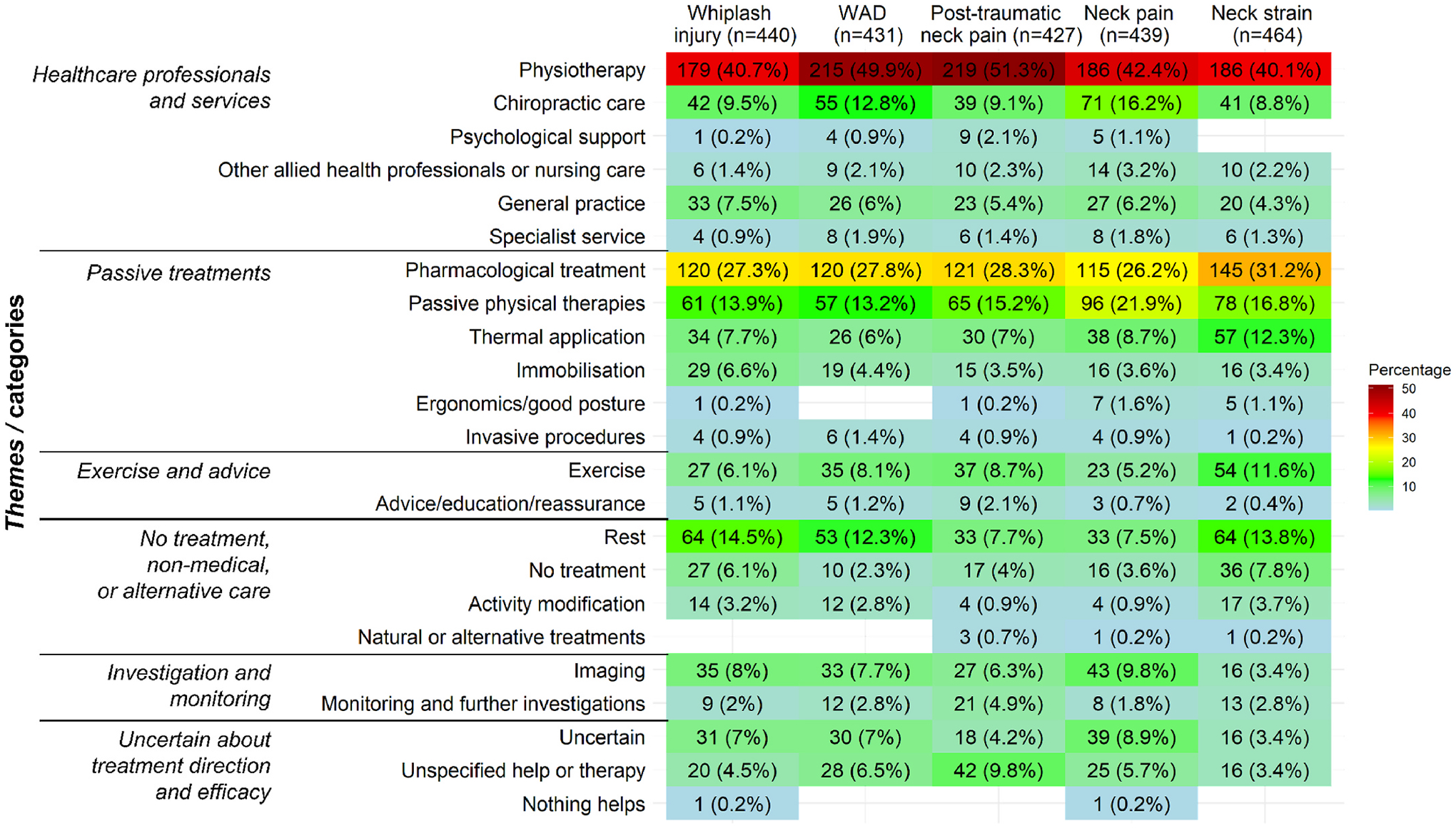
Heatmap shows the frequency of the responses consistent with the themes and categories across label groups for the question: “What health service(s)/treatment(s) (if any) do you think a person with ‘[one of the five labels]’ needs?”. WAD = Whiplash-associated disorder.

Participants often expressed a need for passive treatments that do not require active physical effort, ranging from pharmacological treatment (26.2 %–31.2 %), the most common, to invasive procedures (0.2 %–1.4 %), the least common across groups (Fig. 3). Passive physical therapies such as massage were the second most commonly mentioned passive treatments across groups (13.2 %–21.9 %), especially by participants assigned to *neck pain* (21.6 %).

#### 3.3.3. Exercise and advice

In contrast to passive treatments, ≤12 % of participants expressed a need for exercise and advice (Fig. 3). Exercise was most commonly identified for *neck strain* (11.6 %) and least commonly for *neck pain* (5.2 %). Only 0.4 %–2.1 % of participants across groups expressed the need for advice, education, and/or reassurance.

#### 3.3.4. No treatment, non-medical, or alternative care

Some participants expressed preferences for non-medical or alternative care approaches, including rest and relaxation, activity modification to prevent further injuries, natural or alternative treatments, and/or no formal treatment at all, relying instead on being patient, staying positive, returning to usual activities, and making lifestyle adjustments (Table 3). Among these approaches, rest was most frequently mentioned (7.5 %–14.5 %), particularly among participants assigned to *whiplash injury* (14.5 %), *whiplash-associated disorder* (12.3 %) and *neck strain* (13.8 %). However, many did not specify what they meant by ‘rest’, simply referring to it without further explanations. Labels *whiplash injury* (6.1 %) and *neck strain* (7.8 %) also more frequently elicited thoughts of ‘no treatment’ needed than other labels (2.3 %–4 %).

#### 3.3.5. Investigation and monitoring

Beyond treatments, some participants felt the need for further investigations such as imaging and ongoing symptom monitoring. Participants assigned to *neck strain* (3.4 %) least expressed the desire for imaging compared to those assigned to other labels (6.3 %–9.8 %).

#### 3.3.6. Uncertain about treatment direction and efficacy

A small proportion of participants were uncertain about the necessary health services or treatments (3.4 %–8.9 %), mentioned unspecified help or therapy (3.4 %–9.8 %), and/or felt nothing helps (0.2 %) (Fig. 3). Labels *neck pain* (8.9 %), *whiplash injury* (7.0 %), and *whiplash-associated disorder* (7 %) were more often associated with uncertainty than *post-traumatic neck pain* (4.2 %) and *neck strain* (3.4 %). However, when mentioning the need for help or therapy, participants assigned to *post-traumatic neck pain* (9.8 %) most often did not specify the exact treatments needed, while those assigned to *neck strain* (3.4 %) were least likely to do so.

### 3.4. Question 4:Is there anything confusing about the diagnosis ‘[one of the five labels]’ given by the healthcare provider in the scenario?

For this question, 10 categories and 3 themes were identified: ‘no confusion’, ‘vague and lack of clarity’, and ‘mismatch in diagnostic expectations’ (Table 4).

#### 3.4.1. No confusion

**Fig. 4.**
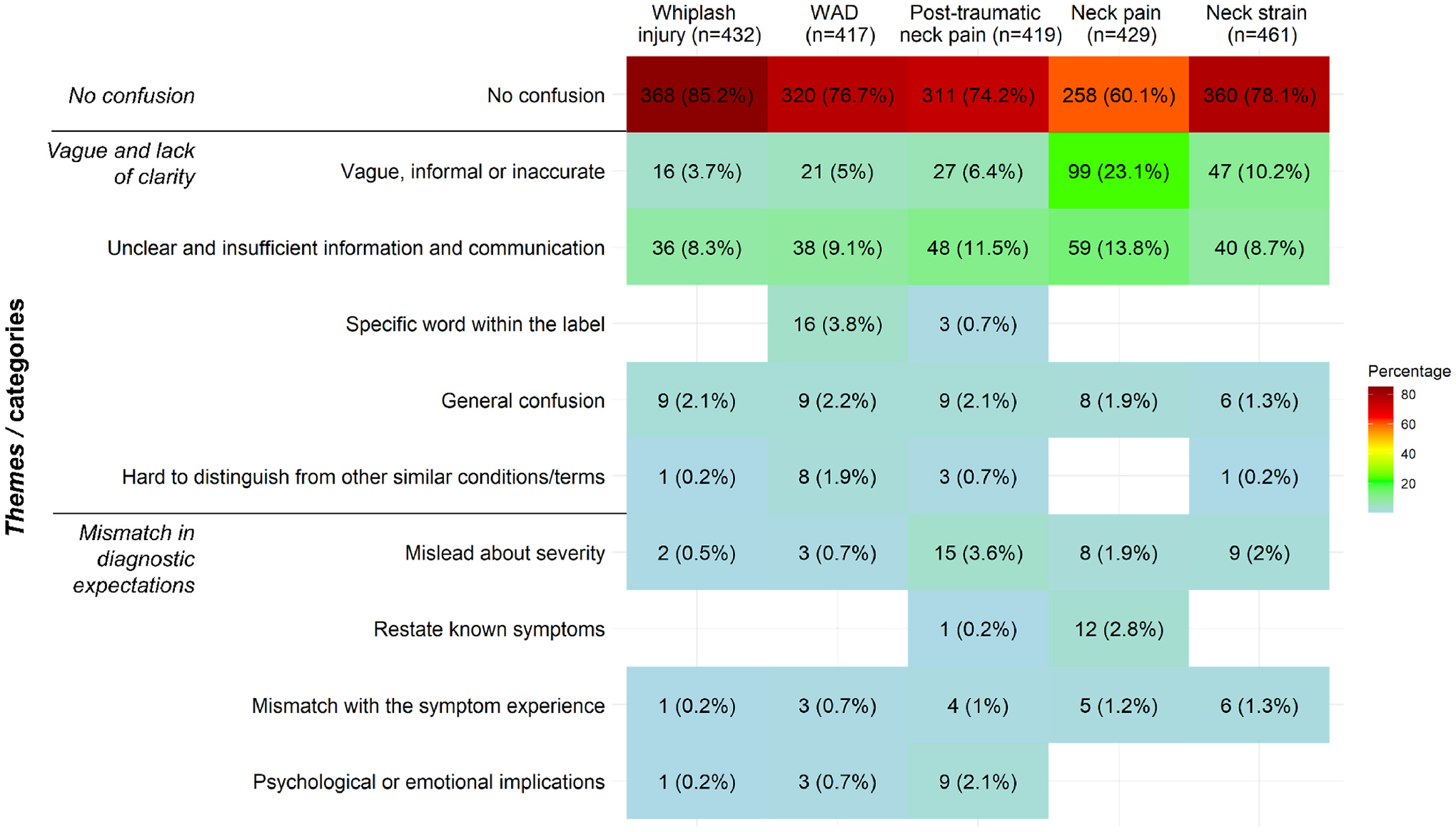
Heatmap shows the frequency of the responses consistent with the themes and categories across label groups for the question: “Is there anything confusing about the diagnosis (‘[one of the five labels]’) given by the healthcare provider in the scenario?”. WAD = Whiplash-associated disorder.

Most participants perceived no confusion about their assigned label (Fig. 4), with this being most common for *whiplash injury* (85.2 %) and least common for *neck pain* (60.1 %).

#### 3.4.2. Vague and lack of clarity

This theme captures responses where participants described their label as vague, informal, or inaccurate, with unclear and insufficient diagnostic information and communication. Some also found specific words within their label confusing and had difficulty distinguishing it from other similar conditions or terms. ‘Vague, informal or inaccurate’ was most frequently mentioned by participants assigned to *neck pain* (23.1 %), followed by *neck strain* (10.2 %), and least by those assigned to *whiplash injury* (3.7 %). A shared confusion source across groups (8.3 %–13.8 %) was unclear or insufficient information and communication about the injury’s cause, nature, severity, expected duration, affected tissue or structure, impact, prognosis, and required treatment plan. This confusion may not have been caused by the label itself, but rather by the message in the vignette, which aimed to reassure participants that their condition is not serious and encouraged them to return to daily activities. For example, a participant mentioned: “*The confusing part is that there is pain without resolution in sight but an encouragement to return to “normal.”*_“_ (Participant 1485, female, aged 40 years). A few participants assigned to *whiplash-associated disorder* (3.8 %) and *post-traumatic neck pain* (0.7 %) expressed negative views about specific words within the label, such as ‘disorder’ and ‘traumatic’. Additionally, 0.2 %–1.9 % participants, particularly those assigned to *whiplash-associated disorder* (1.9 %), found their assigned label could be confused with other terms such as post-traumatic stress disorder (Table 4).

#### 3.4.3. Mismatch in diagnostic expectations

Another source of confusion was a mismatch between participants’ assigned label with their own injury perceptions and diagnostic expectations. Despite infrequent overall (0.5 %–3.6 %), participants assigned to *post-traumatic neck pain* (3.6 %) most often felt this label was misleading about the injury severity and did not align with their symptom experience (Fig. 4). Few participants assigned to *neck pain* (2.8 %) and *post-traumatic neck pain* (0.2 %) felt the labels merely restated known symptoms, without adding value to their understanding of the injury. Furthermore, a few participants assigned to *post-traumatic neck pain* (2.1 %), *whiplash-associated disorder* (0.7 %) and *whiplash injury* (0.2 %) perceived psychological or emotional implications in these labels, feeling a mismatch between physical symptoms and psychological connotations. They also associated these labels with post-traumatic stress, mental disorders and negative emotions such as fear.

## 4. Discussion

Our study found that labels for neck pain after an MVC held varied meanings for participants with or without neck pain from the general population, evoking different feelings and influencing treatment preferences. Overall, compared to other labels, the label *neck strain* was more commonly associated with positive expectations and a preference for exercise, while it was less frequently linked to psychological distress or the need for imaging. *Neck pain* was perceived as the most confusing label, mainly due to its vagueness.

Our findings may explain why, in the quantitative part of our study (Xie et al., 2024), participants assigned to *neck strain* had higher recovery expectations compared to those given other labels including *whiplash-associated disorder*, *neck pain*, and *post-traumatic neck pain*. This content analysis revealed that participants assigned to *neck strain* (17.7 %) were most frequently expressed feelings that the condition was minor and had a good prognosis, particularly compared to participants assigned to *post-traumatic neck pain (6.5 %)* and *neck pain (2.9 %)*. In contrast, negative expectations about symptom severity and prognosis were least commonly mentioned by participants assigned to *neck strain* (4.7 %) compared to those assigned to *post-traumatic neck pain* (16.0 %) and *whiplash-associated disorder* (10.4 %). Similarly, feelings of psychological distress were least commonly linked to the label *neck strain* (7.3 %) compared to other labels, particularly *post-traumatic neck pain* (30.3 %), *neck pain* (13.6 %) and *whiplash-associated disorders* (13.0 %).

Few participants across groups associated their assigned label with legal and financial implications, perceived injustices or questioned the legitimacy of the injury. Whilst these thoughts were nominated more frequently with the labels *whiplash injury* and *whiplash-associated disorder*, the number of participants nominating them was low (Fig. 2). This was surprising considering that compensation related factors (Rydman et al., 2018) and perceived injustice (Sullivan et al., 2009, 2011) about the nature of the MVC injury have been proposed as drivers of poor outcomes after MVC injury. However, as this study was conducted online with participants from the general population, our results may not reflect thoughts of patients seen in clinics.

Labels influenced participants’ treatment needs and preferences. For example, *neck pain* most often prompted need for passive physical therapies (21.9 % versus 13.2 %–16.8 %) and imaging (9.8 % versus 3.4 %–8 %) compared to other labels. In contrast, *neck strain* most frequently evoked a preference for exercise (11.6 % versus 5.2 %–8.7 %) and least frequently for imaging (3.4 % versus 6.3 %–9.8 %) compared to other labels. Current guidelines recommend advice to return to usual activity and exercise as first-line treatment following an MVC musculoskeletal injury, while advising against advanced imaging and some types of passive physical therapies such as electrotherapy, manipulation and needling techniques (State Insurance Regulatory Authority et al., 2023). For other types of passive physical therapies such as acupuncture and massage, current guidelines remain neutral (State Insurance Regulatory Authority et al., 2023). Our findings suggest that the label *neck pain* may encourage people to seek non-recommended care, whereas the label *neck strain* appears to prompt preferences that align better with guideline-recommended treatments. However, exercise (5.2 %–11.6 %) was generally less preferred compared to passive physical therapies (13.2 %–21.9 %) across all groups, contrasting to a similar study of low back pain where exercise was a top treatment preference for different diagnostic labels (41 %) (O’Keeffe et al., 2022b). Additionally, a slightly higher proportion of participants expressed a need for rest (7.5 %–14.5 %) compared to exercise (5.2 %–11.6 %). However, it was unclear if participants meant long-term bed rest or short-term rest because most simply mentioned ‘rest’ without further elaboration. These results suggest that clinicians and policymakers may need to promote the importance of staying active for recovery after an MVC musculoskeletal injury. During clinical consultation, it is important to explore how patients conceptualise rest and their feelings about exercise. This may help inform strategies to best communicate the importance of staying active and align patient expectations with guideline-recommended care.

While most participants found their assigned label clear, around 40 % of those assigned to the *neck pain* label reported confusion, viewing the label as vague and inaccurate. This is likely because this label is less related to mechanisms or causes of pain compared to other labels, and people often expect a diagnosis that explains their pain (Carroll et al., 2016). Some participants also perceived *neck pain* as an informal diagnosis, used when the healthcare provider was unsure of the exact issue. These perceptions may explain why participants assigned to *neck pain* were more likely to seek a second opinion than those assigned to other labels (Xie et al., 2024). Confusion also arose when the label did not match participants’ injury perception and diagnostic expectations. For example, 0.2 %–2.1 % of those assigned to *whiplash injury*, *whiplash-associated disorder*, or *post-traumatic neck pain* found these labels confusing due to the perceived psychological distress associated with them. In contrast, participants viewed neck pain after an MVC purely as a physical injury. This perception of a purely physical injury may explain why most participants in this study felt psychological support as unnecessary, aligning with previous studies, where individuals with pain following an MVC musculoskeletal injury perceived psychology services as irrelevant (Jull et al., 2013; Ritchie et al., 2020), with only 1 % seeking such services (Ritchie et al., 2020).

Across all label groups, participants reported a shared sense of confusion, due to the lack of clear information about the cause, severity, and prognosis of the condition, affected structures and tissues, potential implications and care plans. While this finding may be due to how the vignette was presented to participants, it underscores participants’ desire for not only accurate diagnoses but also clear, detailed, and informative explanations. This desire is consistent with previous research on individuals with non-traumatic neck pain, who commonly express a need for specific and concrete explanations about the origin of their symptoms, comprehensive information about their condition, and clear communication from clinicians regarding effective treatment options (Falsiroli et al., 2022).

Our findings highlight how diagnostic labels may influence the public and patients’ perceptions, recovery expectations, and treatment preferences – an effect consistent with broader literature on the nocebo phenomenon in musculoskeletal pain (Rossettini et al., 2023), where negative language or suboptimal communication by clinicians may contribute to poorer outcomes (Linskens et al., 2023). These findings have important implications for clinical practice, health policy, and future research regarding the use and communication of a diagnostic label for neck pain after an MVC (Table 5). Consistent with our quantitative data (Xie et al., 2024), this content analysis suggests avoiding the label *neck pain*, as it was most likely to cause confusion, elicit feelings of unhappiness/frustration and prompt preference for non-guideline-recommended treatments. *Post-traumatic neck pain* and *whiplash-associated disorder* may also be unfavourable as it more commonly evoked negative expectations and psychological distress compared to other labels such as *neck strain* and *whiplash injury*, partly due to words like ‘traumatic’ and ‘disorder’ within the labels, which some participants linked to post-traumatic stress disorder. In contrast, *neck strain* was generally perceived more positively, aligning with our quantitative findings (Xie et al., 2024). However, some participants (10.2 %) found it vague and inaccurate, which could hinder its uptake as a standard label. On the other hand, *whiplash injury* was perceived as the least confusing and the second least likely to trigger negative expectations and psychological distress, following *neck strain*. Our quantitative data found that the only significant differences between *neck strain* and *whiplash injury* were that the latter slightly increased perceived injury seriousness and led to a slightly greater intention to make a compensation claim (Xie et al., 2024). Considering the small differences of unclear clinical relevance and greater perceived precision, clinicians and patients may prefer to continue adopting the more commonly used label *whiplash injury* over *neck strain*. However, more in-depth qualitative research is needed to confirm the acceptability of various labels for neck pain after an MVC among clinicians, patients and wider stakeholders (e. g. compensation authorities).

Study strengths include a large sample size with high response rates and rigorous methods such as randomisation and concealed allocation. However, several limitations exit. Firstly, the online nature of the study may not accurately reflect people’s actual understanding and perceptions towards different labels in the clinical encounter. Secondly, each label was accompanied by a brief explanation, mirroring the way that a diagnostic label would typically be conveyed to patients in a clinical setting. However, the label explanation may influence participants’ interpretation and perception about the label. Thirdly, the data was collected at a single instance, immediately after labels were assigned. This may bias participants’ responses because people’s understanding, feelings and perceptions may change as they reflect on the label over time. Fourthly, the absence of specific context details about the participant (e.g., prior care experiences) could potentially limit our understanding on participants’ responses, leading to less accurate data coding. Lastly, this study only included English-speaking participants from five high-income countries, limiting the generalisability of the findings to individuals who speak other languages or live in countries with different socioeconomic and cultural contexts (e.g. low- and middle-income countries).

## 5. Conclusions

Compared to other labels, *neck strain* more often elicited positive expectations and a preference for exercise, while less frequently evoking negative expectations, psychological distress and the need for imaging. Participants found the label *neck pain* most confusing and *whiplash injury* least confusing, with confusion stemming from vagueness or a mismatch with diagnostic expectations. When delivering a diagnostic label for neck pain after an MVC, clinicians should consider how people may make sense of the label, emotional responses and treatment preferences it might evoke.

## Figures and Tables

**Table 1:** Themes and categories identified from free-text responses to the question: “What does the term ‘[one of the five labels]’ mean to you?”

Theme	Category	Description	Examples
Injury mechanisms	Quick, forceful, or abnormal movement	Responses suggesting that pain or injury was caused by sudden, abrupt, jerky, jolting, or forceful neck movements.	“Neck injury caused by the sudden extreme jerking of the head back and forth.” *[P314, Female, 75 years, history of neck pain]* “An injury that has happened because of fast movement.” *[P352, Female, 29 years, history of neck pain]* “Quick sudden movements leading to injury” *[P385, Female, 28 years, history of neck pain]*
	Soft tissue (or structure) injury or damage	Response referring to soft tissues (e.g. “muscle”) or body structure, and the injury or damage to soft tissue or structure which sometimes can be invisible. Soft tissue (muscle) injury was most frequently mentioned in this category.	“That there is some damage to the vertebrae” *[P407, Female, 74 years, history of neck pain]* “Soft tissue damage/sprain” *[P317, Female, 69 years, history of neck pain after a motor vehicle crash]* “muscles and/or nerve damage in the neck” *[P372, Female, 74 years, no neck pain history]* “Damage to the neck, non visible” *[P2221, Male, 28 years, no neck pain history]*
	Accident or trauma	Responses suggesting that pain/injury was related to car accident or trauma.	“When I hear those words, it makes me think of neck and back pain from being involved in an accident.” *[P11, Female, 46 years, history of neck pain]* “Pain that is caused by trauma rather than injury” *[P73, Female, 21 years, history of neck pain]* “Getting injured by a car” *[P177, Female, 18 years, history of neck pain]*
	Delayed manifestation of an event	Responses referring to the delayed onset of pain or other symptoms after an event (e.g. injury, incidence, accident, or trauma). This delay ranged from a few hours to days, weeks, or even longer after the event.	“There was trauma that did not become bothersome until after the event which caused it.” *[P1212, Female, 37 years, history of neck pain after a motor vehicle crash]* “Pain caused a bit later as a reaction to the crash” *[P1960, Female, 66 years, no neck pain history]* “The pain was not immediately felt” *[P2029, Female, 61 years, no neck pain history]*
	Whiplash	Responses referring that symptoms were caused or associated with whiplash or perceiving the diagnostic label as an alternative name for whiplash.	“It means symptoms you have were received due to whiplash” *[P114, Female, 71 years, history of neck pain]* “Similar to whiplash” *[P1164, Female, 58 years, history of neck pain]* “To me it is just whiplash” *[P2156, Male, 42 years, no neck pain history]*
	Incorrect posture or other causes	Responses that associated pain/injury with an incorrect posture (e. g. funny posture during sleep) or other causes such as diet.	“Many times, the foods we have eaten go and deposit in the neck causing tightness and pain. A good fasting can remove many of the tightness and pain (my own experience)” *[P1006, Male, 37 years, history of neck pain]* “Not a lot, maybe sleeping funny in bed, maybe from sitting at a desk all day” *[P1854, Female, 61 years, no neck pain history]*
Physical symptoms and function	Pain and associated symptoms	Responses in which the label evoked associations with pain, ouch, hurt, fatigue, stiffness, tightness, spasm, discomfort, aching, soreness, aching, tenderness, tension, numbness, tingling, headache, dizzy, tiredness, fatigue, nausea, fainting, swelling, other physical symptoms and sensation such as headache, migraine, and dizziness, without further references to the severity, expected symptom duration, or impact on daily activities and quality of life.	“Pain felt mostly in the neck and immediate surrounding area.” *[P90, Female, 65 years, no neck pain history]* “Ache and pain in the neck area which radiates down the arms and can cause headache” *[P173, Female, 26 years, history of neck pain]* “Main within the neck, causing headaches and dizziness” *[P187, Female, 19 years, history of neck pain]*
	Interference with daily activities	Responses that associated the diagnostic label with movement restriction, interference with daily activities, or disruption of overall wellbeing and quality of life.	“hurting when you do certain things.” *[P1633, Female, 49 years, history of neck pain]* “Inability to move and function as normal” *[P76, Female, 27 years, history of neck pain after a motor vehicle crash]* “Totally inconvenience.” *[P1044, Female, 55 years, history of neck pain]* “EITHER DOFT TISSUE DAMAGE OR NERVE DAMAGE IMPACTING QUALITY OF LIFE” *[P1908, Male, 66 years, no neck pain history]*
Divergent expectations	Negative expectations	Responses that related the diagnostic label with negative expectations about symptoms or prognosis. These negative expectations included beliefs that the injury, pain, or other symptoms are serious, bad, terrible, horrible, dangerous, unbearable, complex or recuring, chronic and permanent. This theme also included responses that expressed a sentiment of helplessness, believing that there is nothing or little that can be done to alleviate the pain and associated symptoms.	“Continual pain for months on end. Neck never being the same. Numbness, tingling and pain on and off for the rest of my life.” *[P139, Female, 61 years, history of neck pain]* “Chronic pain of the neck” *[P1792, Male, 38 years, no history of neck pain]* “Bad whiplash” *[P1104, Female, 22 years, history of neck pain]* “Neck pain means it is unbearable pain that would not go away” *[P2121, Female, 74 years, no neck pain history]* “Severe pain and stiffness in the neck area.” *[P2194, Female, 63 years, no neck pain history]*
	Positive expectations	Responses that related the diagnostic label with positive expectations about symptoms or prognosis. These positive expectations included beliefs that the injury, pain, or other symptoms were non-serious, minor, not a cause for concern, not bad, not dangerous, not significant, or temporary and non-chronic. This theme also reflected expectations about an easy or quick recovery, with or without treatments.	“It is a pain that will go away on its own” *[P230, Female, 78 years, history of neck pain]* “Sounds like somethings that is temporarily and quickly” *[P243, Female, 290 years, history of neck pain]* “A non-serious condition that doesn’t require medical attention, should resolve on it’s own after some time” *[P563, Female, 25 years, history of neck pain]* “Very minor injury to the neck and/or shoulder” *[P1494, Female, 20 years, history of neck pain]*
Psychosocial implications	Psychological distress	This theme highlights responses associate the diagnostic label with psychological distress such as fear, stress, irritation, frustration, anxiety, post-traumatic syndrome, or post-traumatic stress disorder. Some participants also perceived the injury as a psychological issue or a result of stress, often related to an accident or traumatic event.	“It seems to mean that there may be a mental aspect to the neck pain.” *[P769, Male, 70 years, history of neck pain]* “I think it is an unwise choice of phrase as it brings forward an image of mental distress …” *[P1826, Male, 54 years, no neck pain history]* “Stress mentally causing neck pain after the accident” *[P2140, Male, 57 years, no neck pain history]*
	Legal or financial implications	Responses that related the diagnostic label with legal or financial implications, including any reference to compensation claims or insurance, legal proceedings, financial accountability, or possible financial consequences (e.g. through sick leave, healthcare bills).	“Law suit” *[P489, Male, 64 years, history of neck pain]* “A lot of people use this as a way of making money.” *[P1936, Female, 58 years, no neck pain history]* “Working in insurance claims, it has historically been very easy for people to make claims” *[P1727, Female, 37 years, no history of neck pain]*
Management strategies	Treatment/investigation	Responses that referred to a need for treatments (e.g. medications, physiotherapy, or chiropractic care) for recovery, or further investigations (e.g. MRI) to rule out more serious damage.	“. I would expect my doctor to do further testing to rule out more serious damage.” *[P884, Female, 68 years, history of neck pain after a motor vehicle crash]* “An ache that necessitates taking pain killers” *[P1922, Female, 48 years, no history of neck pain]*
	Non-medical strategies	Responses that referred to a need for time, patience, rest/relaxation, taking things easy, being cautious, cutting down or returning to usual activity.	“Need to rest & relax” *[P1625, females, 26 years, current neck pain]* “… It is not serious but requires the person to try their best to resume their normal daily activities.” *[P1405, Male, 52 years, history of neck pain]*
Diagnostic clarity and significance	Vague or inaccurate diagnosis	Responses in which the diagnosis label was perceived as vague, generic, made-up, or inaccurate, or reflected the healthcare provider’s lack of understanding of the injury.	“It means the GP has no idea what is casing the issue but wants me to feel they have listened.” *[P422, Female, 48 years, history of neck pain]* “It sounds like a generic term, but not a real medical diagnosis.” *[P644, Female, 49 years, history of neck pain]*
	Uncertainty or indifference	Responses that expressed uncertainty due to lack of clarity or knowledge about the severity, impact or prognosis of the injury, as well as feeling of indifference or perceived insignificance towards the diagnostic label.	“Anything from a stiff neck to scoliosis or actual short term to long term damage that impacts normal daily physical activities and sleep.” *[P999, Female, 63 years, history of neck pain]* “Pain in my neck that is either continuous or sporadic” *[P2095, Female, 37 years, no neck pain history]* “Not sure” *[P207, Female, 21 years, history of neck pain]* “It doesn’t mean much to me” *[P296, Female, 18 years, history of neck pain]*

Note: P = Participant.

**Table 2:** New themes identified from free-text responses to the question: “When you hear the term ‘[one of the five labels]’, what words or feelings does this make you think of?”

Theme	Category	Description	Examples
Negative emotions	Unhappiness/frustration	Responses that referred to feeling unhappy, unpleasant (e.g. bad, not nice, awful), sad, sorrow, fed up, bothersome, unfortunate, annoyed, niggling, angry, irritated, cranky, aggravation, frustrated, hopeless, nuisance, impatience, aggressive, miserable, grinding or expressing that the situation ‘sucks.’	“Saddened by pain” *[P245, Female, 30 years, history of neck pain]* “I would feel frustrated to receive such a vague diagnosis” *[P844, Female, 70 years, history of neck pain after a motor vehicle crash]* “Aggravation, particularly when I was not at fault for the conditions that caused the injury.” *[P317, Female, 69 years, history of neck pain after a motor vehicle crash]*
	Agony/suffering/disorder	Responses which referred to agony, suffering, or disorder.	“pain agony trauma” *[P570, Female, 28 years, history of neck pain]* “Suffering” *[P2216, Female, 65 years, no neck pain history]*
	Injury legitimacy	Responses referring to dishonest, fraudulent, erroneous insurance claim or an injury that is perceived to be imaginary, unreal, non-existent, or hard-to-verify.	“made up” *[P986, Female, 53 years, history of neck pain]* “People who make erroneous insurance claims” *[P1880, Female, 59 years, no neck pain history]*
	Dismissal	Responses which referred to feeling dismissed, not being taken seriously, or their health and pain being overlooked.	“That I’m not being taken seriously and that my health and pain is being overlooked” *[P805, Female, 37 years, history of neck pain]* “It makes me think of pain and being ignored by doctors/not taken seriously. This creates hopelessness and makes me ask myself of I’m crazy/imagining that pain.” *[P1171, Non-binary/Gender-Fluid, 38 years, current neck pain]*
	Regret	Responses referring to the feeling of regret	“… regret …” *[P700, Female, 77 years, history of neck pain]* “The term makes me feel regretful and angry about the accident.” *[P1589, Female, 65 years, history of neck pain]*
	Injustice	Responses which related the diagnostic label with thoughts about being victimised and feeling of unfair.	“Car accident victim” *[P873, Female, 43 years, history of neck pain]* “It generally makes me feel that someone has been injured as a result of another driver’s careless or wreckless driving.” *[P2177, Male, 54 years, no neck pain history]*
Empathy, validation and hope	Empathetic resonance	Responses in which the diagnostic label resonated with participants on an empathetic level, evoked memories of their own experiences and feelings of sympathy for people with a similar injury.	“Feeling bad if it’s happening to someone else …” *[P709, Female, 55 years, history of neck pain]* “I feel sympathy for anyone suffering from neck strain” *[P1431, Male, 48 years, history of neck pain]*
	Feeling validated	Responses which described feelings of being believed or validated or feeling of relieved.	“I think I would feel relieved to have a diagnosis.” *[P908, Female, 29 years, history of neck pain]* “It makes me feel like I understand why there is now bad pain in the neck area. It also makes me feel like I’m not imagining the pain, that it’s real, but not serious.” *[P1405, Male, 52 years, history of neck pain]*
	Hope for recovery	Responses that referred to participants’ hopes for the injury being short-term, going away or eventually recover.	“makes me hop it goes away so can have a normal life” *[P323, Male, 82 years, history of neck pain after a motor vehicle crash]* “HOPEFULLY NOT LONG LASTING” *[P2179, Female, 72 years, no history of neck pain]*

Note: P = Participant.

**Table 3:** Themes and categories identified from free-text responses to the question: “What health service(s)/treatment(s) (if any) do you think a person with ‘[one of the five labels]’ needs?”

Theme	Category	Description	Examples
Healthcare professionals and services	Physiotherapy	Responses which specifically referred to physiotherapist, PT, physiotherapy, or physical therapy.	“Possible physiotherapy” *[P150, Male, 79 years, history of neck pain]* “Physical therapy” *[P413, Female, 33 years, history of neck pain]*
	Chiropractic care	Responses which specifically referred to chiropractor or chiropractic care.	“Possible a chiropractor” *[P25, Female, 76 years, history of neck pain]* “I think they need to be referred to the chiropractor” *[P308, Female, 38 years, history of neck pain]*
	Psychological support	Responses that referred to psychologists, psychology, mental health counselling, and support for managing stress, emotional, and psychological issues such as relaxation techniques, mental exercise and cognitive physical therapy.	“… help to deal with mental, emotional effects” *[P435, Female, 72 years, history of neck pain after a motor vehicle crash]* “Meet with a psychologist.” *[P769, Male, 70 years, history of neck pain]*
	Other allied health professionals or nursing care	Responses which referred to nurses and other allied health professionals, excluding physiotherapists and chiropractors, such as osteopaths, pharmacists, exercise physiologists, and occupational therapists, or the care they provide.	“Osteopath” [P513, Female, 27 years, history of neck pain] “Pharmacist, Nurse” *[P2073, Female, 48 years, no history of neck pain]*
	General practice	Responses which specifically referred to a doctor, general doctor, general practitioner (GP), family doctor or the care they provide.	“General doctor” *[P89, Male, 23 years, history of neck pain]* “family doc first” *[P276, Female, 38 years, history of neck pain]*
	Specialist service	Responses which specifically referred to seeing a specialist including pain management specialist, neurologist, specialist surgeon, orthopaedic doctor, psychiatrist, emergency or urgent care and treatment.	“…, a specialist surgeon on the neck” *[P115, Female, 67 years, no history of neck pain]* “Maybe an orthopaedic doctor if the pain persists” *[P1439, Female, 70 years, history of neck pain after a motor vehicle crash]*
Passive treatments	Pharmacological treatment	Responses referred to pharmacological treatments, including oral medications (e.g., tablets, pills) and topical medications (e.g., anti-inflammatory creams). Examples include muscle relaxants, paracetamol, ibuprofen, pain relievers, anti-inflammatories, over-the-counter medications, steroids, sleeping aids, anti-anxiety medications, and topical pain relief gels and creams.	“Topical pain relief” *[P504, Female, 64 years, history of neck pain]* “… Put medical creams or serums around the neck to be healed” *[P1584, Female, 48 years, no neck pain history]* “medication like muscle relaxants, anti anxiety meds” *[P1658, Female, 74 years, no neck pain history]*
	Passive physical therapies	Responses which referred to modalities applied to the person without active physical effort from the person, such as massage (*most frequently mentioned)*, electrotherapy (e.g. TENS machine), ultrasound therapy, acupuncture, dry needling, manipulation, muscle rub, neck adjustment.	“… Lazer treatment in my case …” *[P939, Female, 55 years, history of neck pain after a motor vehicle crash]* “Massage or acupuncture” *[P1283, Male, 60 years, history of neck pain]* “… Maybe some new methods like dry needling” *[P718, Male, 18 years, history of neck pain]*
	Thermal application	Responses which referred to heat (e.g. heat packs/bag, heat/hot compression, warm bath or clothes) and/or cold (e.g. ice or cool packs) application.	“Heat compression” *[P81, Female, 32 years, history of neck pain]* “Ice pack” *[P1710, Non-binary/gender fluid, 30 years, no neck pain history]*
	Ergonomics/good posture	Responses referring to ergonomic strategies and maintaining a good posture	“… neck pillows, better posture” *[P219, Non-binary/gender fluid, 18 years, no neck pain history]* “pain reducing equipment(… pillows)” *[P1755, Female, 52 years, no neck pain history]*
	Immobilisation	Responses which suggested a need for immobilisation through various devices such as a neck brace, an isolation brace, a c-collar, a neck cast, a neck band, or even tape.	“… Tape to keep everything in shape” *[P302, Female, 24 years, history of neck pain]* “Wear a neck collar.” *[P1249, Female, 62 years, no neck pain history]*
	Invasive procedures	Responses which referred to invasive treatments including surgery, steroid or anti-inflammatory shots, and injection of steroids, cortisone, or lidocaine	“Injections of steroids in the neck” *[P139, Female, 61 years, history of neck pain]* “could need surgery” *[P843, Male, 75 years, history of neck pain]*
Exercise and advice	Exercise	Responses which referred to (gentle) general and therapeutic exercise, stretching, (gentle) movement, (neck muscle) strengthening training, physical activity, yoga, aquatics (e.g. swimming, hydrotherapy).	“Neck movements” *[P838, Male, 21 years, history of neck pain]* “Daily exercises to stretch and strengthen the neck muscles” *[P1883, Female, 32 years, no neck pain history]* “Yoga *[P1224, Female, 27 years, history of neck pain]*
	Advice/education/reassurance	Responses that referred to seeking advice, education and/or reassurance	“Instructions on how to care for the injury” *[P637, Male, 51 years, history of neck pain]* “Health care advice” *[P2108, Male, 40 years, no neck pain history]*
No treatment, non-medical, or alternative care	Rest	Responses referring to rest, relaxation, giving up activities, or time off work.	“Needs to lie down and rest” *[P210, Male, 36 years, history of neck pain]* “Rest” *[P535, Female, 50 years, history of neck pain]* “… inactivity …” *[P999, Female, 63 years, history of neck pain]*
	No treatment	Responses in which participants felt no formal medical treatment was needed, just be patient, stay positive, return to usual activities, allow time for recovery, or making lifestyle adjustments (e.g., drinking water, sleep).	“Return to normal activities” *[P565, Male, 68 years, no neck pain history]* “None really.” *[P859, Female, 39 years, history of neck pain]* “To drink water, sleep” *[P1242, ‘other’ gender, 29 years, history of neck pain]*
	Activity modification	Responses referring to being careful, restricting or reducing certain movements and activities to avoid further injury or pain aggravation. Examples included reducing or avoiding strenuous activities, sudden or repetitive movements, physically exertive activities, daily tasks like driving that were seen as additional strain and keeping away from stress.	“no physically exertive activity” *[P108, Male, 71 years, history of neck pain]* “No driving since this will cause further strain …” *[P1497, Female, 58 years, history of neck pain]* “Just don’t make it worse” *[P1860, Female, 38 years, no neck pain history]*
	Natural or alternative treatments	Responses which referred to natural remedies, naturopath, alternative therapies, natural plant herb-based therapies.	“… all natural remedies” *[P607, Female, 45 years, history of neck pain]* “… alternative therapies to help treat the pain.” *[P729, Female, 44 years, current neck pain]*
Investigation and monitoring	Imaging	Responses which referred to the need for imaging, e.g. MRI, CAT scan, X-ray, CT, ultrasound.	“I would say an exam, an x-ray and/or a CT or MRI.” *[P105, Female, 48 years, history of neck pain]* “A second opinion. Physiotherapist for an actual diagnosis …” *[P805, Male, 37 years, current neck pain]* “Monitoring of condition - reassessment if does not resolve …” *[P1765, Male, 65 years, no neck pain history]*
	Monitoring and further investigations	Responses which referred to the need for ongoing monitor, check-ups, follow-up visits, a more accurate diagnosis, seeking a second opinion, and undergoing blood tests or further comprehensive tests that may not be specifically defined.	“Further exams and more testing.” [P727, *Female, 69 years, history of neck pain]* “A second opinion. Physiotherapist for an actual diagnosis …” [P805, *Female, 37 years, history of neck pain]* “Ongoing monitoring.” *[P1746, Male, 63 years, no neck pain history]*
Uncertain about treatment direction and efficacy	Uncertain	Responses in which participants indicated having no ideas, uncertainty or found it difficult to be definitive about what health services they perceived as necessary because this could vary based on specific circumstances or conditions related to the individual’s injury or pain.	“I am unsure the treatment or services that I would need.” *[P554, Female, 44 years, history of neck pain]* “It depends on the cause” *[P610, Female, 22 years, history of neck pain]* “I do not know as I am not a medical professional.” *[P1739, Female, 42 years, no history of neck pain]*
	Unspecified help or therapy	Responses that referred to therapy, pain management, medical treatment, or professional help, without specifying the exact nature of the treatments they needed.	“Anything to help with neck pain” *[P845, Male, 32 years, history of neck pain after a motor vehicle crash]* “Pain management, therapy” *[P1456, Female, 25 years, history of neck pain after a motor vehicle crash]* “support and treatment” *[P2000, Male, 75 years, no neck pain history]*
	Nothing helps	Responses which referred to nothing as being helpful to treating the condition.	“Nothing unfortunately” *[P1005, Female, 60 years, history of neck pain]* “Unfortunately nothing helps” *[P1794, Female, 58 years, no neck pain history]*

Note: P = Participant.

**Table 4:** Themes and categories identified from free-text responses to the question: “Is there anything confusing about the diagnosis (‘[one of the five labels]’) given by the healthcare provider in the scenario?”

Theme	Category	Description	Examples
No confusion	No confusion	Responses that indicated no confusion related to the diagnostic label.	“No” *[P1, Female, 35 years, history of neck pain]* “Not that I can see.” *[P2220, Female, 41 years, no neck pain history]* “Nothing is confusing.” *[P2229, Male, 61 years, no neck pain history]*
Vague and lack of clarity	Unclear and insufficient information and communication	Responses that indicated confusions due to lack of information about cause and nature of the injury, symptom severity, the expected duration of symptoms, the specific tissue or structure affected (e.g. muscles, tendons, nerves), injury impact and prognosis. Participants also expressed dissatisfaction with the lack of a thorough investigation to fully understand their injury, the perceived absence of a clear treatment plan, and the uncertainty surrounding the advice to return to normal activities as soon as possible. However, it may be the diagnostic label itself and/or the accompanying message aimed to reassure participants contribute to this confusion and dissatisfaction.	“Yes and no. What will the future be after this injury.” *[P68, Female, 29 years, history of neck pain after a motor vehicle crash]* “no a lot of concrete information/advice about the neck pain issue” *[P175, Female, 37 years, history of neck pain]* “When the doctor said go about your normal activities.” *[P345, Female, 65 years, history of neck pain]* “The confusing part is that there is pain without resolution in sight but an encouragement to return to “normal.”” *[P1485, Female, 40 years, history of neck pain]* “I would wonder what the extent of the injury is and if it is only short term or could have long term implications.” *[P781, Female, 33 years, history of neck pain]*
	Vague, informal or inaccurate	Responses in which the diagnostic label was perceived as vague, overly broad, general, inaccurate, dismissive, informal, lacking technical terminology, and not useful. It also included the perception of the diagnostic label as a ‘catch-all’ or back-up term that healthcare providers used when they were unsure of the exact problem.	“Very vague” *[P24, Female, 26 years, history of neck pain]* “It seems like a backup explanation” *[P465, Male, 76 years, history of neck pain]* “It seems like a ’catch all’ diagnosis because they don’t really know what’s wrong” *[P1756, Female, 50 years, no neck pain history]* “Not official medical” *[P388, Male, 35 years, history of neck pain]*
	Specific word within the label	Responses in which participants indicated confusion related to a specific word within the diagnostic label: ‘disorder,’ ‘associated’ and ‘traumatic’. Participants questioned the rationale behind using these specific words as part of the diagnostic label.	“Disorder makes me think it might not go away.” *[P1003, Female, 29 years, history of neck pain]* “Just the “traumatic” part. The neck is a sensitive body part. *[P309, Female, 21 years, history of neck pain]* “Yes the words associated disorder.” *[P1639, Female, 61 years, no neck pain history]*
	General confusion	Responses in which participants expressed a general confusion about the labels without providing specific reasons, questioned and sought clarification about the diagnostic label.	“What is it?” *[P427, Female, 18 years, history of neck pain]* “Why the term” *[P589, Female, 32 years, history of neck pain]* “Yes. Because I don’t understand the term.” *[P777, Female, 18 years, history of neck pain]*
	Hard to distinguish from other similar conditions/terms	Responses that indicated confusion about the diagnostic label in comparison with other more familiar labels or conditions such as concussion, head injury, ‘whiplash’ or post-traumatic stress disorder.	“people could mistaken it as concussion or head injury” *[P112, Male, 72 years, history of neck pain]* “Is it whiplash or just something similar to whiplash?” [P198, Female, 28 years, history of neck pain] “Could be confused with PTSD” *[P1253, Male, 54 years, history of neck pain]*
Mismatch in diagnostic expectations	Mislead about severity	Responses which expressed the diagnostic label either overstated or understated the injury severity.	“sounds very serious adding post-traumatic” *[P1621, Female, 26 years, history of neck pain]* “Yes, sounds more serious than it may be.” *[P1622, Male, 34 years, history of neck pain]* “Its misleading its like the physician is downplaying the injury” *[P30, Female, 58 years, history of neck pain]* “yes, it understates possible long term problems” *[P1014, Male, 71 years, history of neck pain after a motor vehicle crash]*
	Restate known symptoms	Responses which expressed the diagnostic label was merely a reiteration of the symptom they were already aware of, making it over simplified, redundant, or unhelpful.	“Only that it simply tells me what I already knew, namely that I have a sore neck.” *[P769, Male, 70 years, history of neck pain]* “Well if I go to a healthcare provider with neck pain, and they do an assessment, and then tell me I’ve got neck pain, then they are telling me what I have already told them. It’s not a diagnosis, it’s a symptom. And it’s symptom that I am well aware of, otherwise I wouldn’t be there in the first place.” *[P1754, Male, 68 years, no neck pain history]*. “It’s not really a diagnosis because I already knew I had neck pain” *[P2157, Male, 69 years, no neck pain history]*
	Mismatch with the symptom experience	Responses in which participants felt that the diagnostic label did not match the symptoms they experienced, or the body of areas affected.	“Yes because it doesn’t explain why my shoulder would also hurt” *[P833, Female, 33 years, history of neck pain]* “It did not only affect the neck but the head and shoulders too. Maybe post traumatic upper body pain.” *[P1764, Female, 51 years, no neck pain history]* “Feels like it doesn’t match the symptoms presented” *[P1776, Female, 29 years, no neck pain history]*
	Psychological or emotional implications	Responses in which participants perceived the diagnostic label as suggesting a mental or psychological issue rather than a physical one, and participants associated the label with negative emotions like post-traumatic stress, mental disorders, and fear.	“I think it sounds really frightening. Nobody wants to be whipped and nobody wants to be lashed, and both of those sound like they could have severe complications.” *[P129, Female, 78 years, history of neck pain after a motor vehicle crash]* “Yes don’t like that name. Reminds me of post traumatic stress.” *[P915, Female, 42 years, history of neck pain]* “Post-traumatic has a link to mental distress and a more serious issue, it is an unwise choice for this” *[P1855, Male, 66 years, no neck pain history]*

Note: P = Participant.

**Table 5:** Summary of study implications for clinical practice, health policy, and research.

Domain	Study implication
Clinical practice	Clinicians should be mindful of the diagnostic labels they use when communicating with patients with neck pain following a motor vehicle crash, and take the opportunity to explain these labels using reassuring language to reduce unhelpful beliefs. Compared to other labels, the label *neck strain* is generally viewed more positively but requires clear explanation to limit potential confusion.
Health policy	It may be important to include labelling and communication considerations in clinical guidelines. This could extend to resources beyond clinical interactions (e.g. brochures, government or clinic websites), as doing so might positively influence how neck pain after a motor vehicle crash is discussed more broadly.
Research	Further research is needed: To explore the acceptability and impact of diagnostic labels for neck pain following a motor vehicle crash among diverse stakeholders (e.g., patients, clinicians, and compensation authorities). To identify best practice for communicating diagnostic labels in ways that support patient recovery and reduce confusion.

## Data Availability

The complete dataset for this study is available upon request from the corresponding author. The data may only be reused with the corresponding author’s permission and after obtaining appropriate ethical approvals.

## Box 1 The scenario of neck pain following a road traffic crash.

Road traffic crashes occur commonly and can happen to anyone. This next section describes a person with neck complaints following a road traffic crash who goes to a health care provider.

We want you to **put yourself into this scenario** and **do your best** to imagine that you are the person having neck complaints following a road traffic crash.

After reading it, you will be asked several questions. Please do your best to answer them based on this imagined scenario.

### Please read the following scenario carefully.

#### You have neck complaints following a road traffic crash

Two weeks ago, you were stopped at traffic lights in your car, when another car hit you from behind. You estimated the car to be travelling at around 20 km/hour (~12 miles/hours) before it hit you. Your head did not hit anything, but the force of the crash caused your head and neck to move forward and then back again. You had slight pain in your neck immediately after the crash. You attended the hospital Emergency Department, where they took an x-ray of your neck which confirmed there was no major injury. You were discharged with a prescription for pain medicine.

It is now two weeks after your injury:

- You now have pain on the right side of your neck which you rate as 4–5/10 in severity (*where 0 is no pain at all and 10 is the worst possible pain*).
- You can move your neck, but the range is very limited by pain, and you feel ‘stiff’ and ‘tight’.
- When you move your neck to the right to look over your shoulder, the pain spreads to your right shoulder.
- You also have a headache at the base of your skull and tingling in your arms that comes and goes.
- You have trouble getting comfortable at night due to your neck pain and this interrupts your sleep.
- You do NOT have nausea, vomiting, dizziness, or blurred vision.

#### You visit a health care provider (e.g., General Practitioner/Physician or Physiotherapist/Physical therapist)

Your health care provider asks you questions about your neck pain, the car crash, and some health questions.

Your health care provider does a detailed physical examination, which involves:

- Looking at your neck and posture to check if there are any abnormalities
- Examining your neck and shoulder blades to check for painful areas
- Checking whether you can move your neck in certain directions and whether this causes pain
- Checking your reflexes, strength, and sensation in your arms

#### After this, your health care provider tells you:

“You have [*one of the five labels*]. [*Brief explanation of the label*]”.

“I am confident that there is not anything seriously wrong with your neck. It is important for you to return to your daily activities as best as you can. Together we will develop a plan for your treatment.”

Note: This vignette, including the accompanying image, was originally published in the *Journal of Orthopaedic & Sports Physical Therapy* (Xie et al., 2024) and is re-used here with permission.
